# Ventricular Arrhythmias in Left Ventricular Assist Device Patients—Current Diagnostic and Therapeutic Considerations

**DOI:** 10.3390/s24041124

**Published:** 2024-02-08

**Authors:** Laura Załucka, Ewa Świerżyńska, Michał Orczykowski, Krzysztof Dutkowski, Jarosław Szymański, Jarosław Kuriata, Rafał Dąbrowski, Piotr Kołsut, Łukasz Szumowski, Maciej Sterliński

**Affiliations:** 1 Department of Cardiac Surgery and Transplantology, National Institute of Cardiology, 42 Alpejska Street, 04-628 Warsaw, Poland; lzalucka@ikard.pl (L.Z.); jszymanski@ikard.pl (J.S.); pkolsut@ikard.pl (P.K.); 2 1st Department of Arrhythmia, National Institute of Cardiology, 42 Alpejska Street, 04-628 Warsaw, Poland; 3 Doctoral School, Medical University of Warsaw, 61 Zwirki I Wigury Street, 02-091 Warsaw, Poland; 4 Department of Coronary Artery Disease and Cardiac Rehabilitation, National Institute of Cardiology, 42 Alpejska Street, 04-628 Warsaw, Poland

**Keywords:** ECG, heart failure, left ventricular assist device, arrhythmia, Holter ECG

## Abstract

Left ventricular assist devices (LVAD) are used in the treatment of advanced left ventricular heart failure. LVAD can serve as a bridge to orthotopic heart transplantation or as a destination therapy in cases where orthotopic heart transplantation is contraindicated. Ventricular arrhythmias are frequently observed in patients with LVAD. This problem is further compounded as a result of diagnostic difficulties arising from presently available electrocardiographic methods. Due to artifacts from LVAD-generated electromagnetic fields, it can be challenging to assess the origin of arrhythmias in standard ECG tracings. In this article, we will review and discuss common mechanisms, diagnostics methods, and therapeutic strategies for ventricular arrhythmia treatment, as well as numerous problems we face in LVAD implant patients.

## 1. Introduction

Left ventricular assist devices (LVAD) are a heterogeneous class of devices increasingly used in the treatment of advanced heart failure [1], with more than 50,000 LVADs having been implanted over the prior decades worldwide. Currently, their application includes patients for whom standard pharmacological treatment and cardiac implantable electronic devices (CIEDs) do not yield satisfactory results, with the only remaining viable option being orthotopic heart transplantation (OHT). Due to a shortage of suitable donors, LVADs serve as a bridging method (BTT—bridge to transplant) allowing patients time to wait for donor availability and to improve and optimize their general condition prior to OHT surgery. These devices are also being used more frequently as a destination therapy (DT) [2]. Providing care for LVAD patients requires the establishment of a multidisciplinary mechanical support team comprising experienced cardiologists, electrophysiologists, cardiothoracic surgeons, nurses, and a LVAD coordinator [3]. This multidisciplinary team must be capable of taking a holistic approach in optimizing the parameters of mechanical circulatory support and treating any complications that may arise as well as treating the underlying condition of cardiac failure according to current medical standards based on etiology. In our experience, many complications from mechanical circulatory support, including LVAD therapy, arise from the interplay between hemodynamic function and cardiac electrical activity. In particular, ventricular arrhythmias serve as a good disease model to demonstrate the interconnections between the pathophysiology of hemodynamic and electrical function as well as to showcase therapeutic strategies that uniquely apply to this patient group. Thus, in this article, we will specifically focus on, discuss, and review common mechanisms, diagnostics methods, and management strategies for ventricular arrhythmia in LVAD implant patients.

### 1.1. An Overview of Design Principles and Functional Mechanics of Left Ventricular Assist Devices

**Figure 1:**
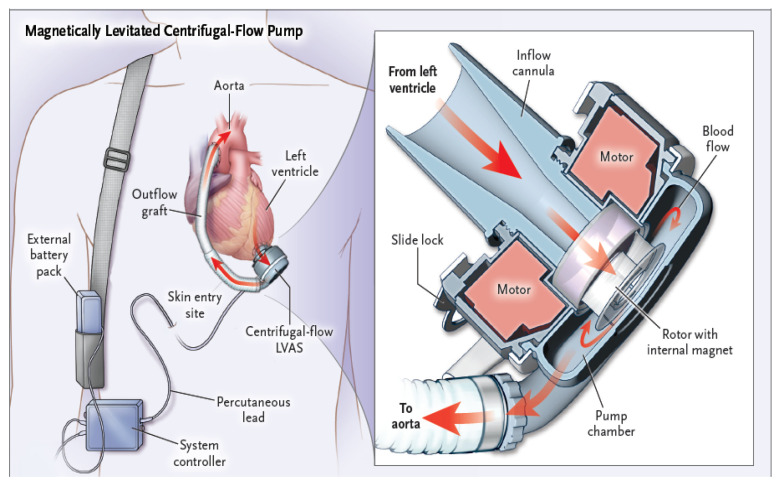
Centrifugal pump LVAD. The illustration depicts the structural schematic of a centrifugal pump and its placement within the thoracic cavity.

**Figure 2:**
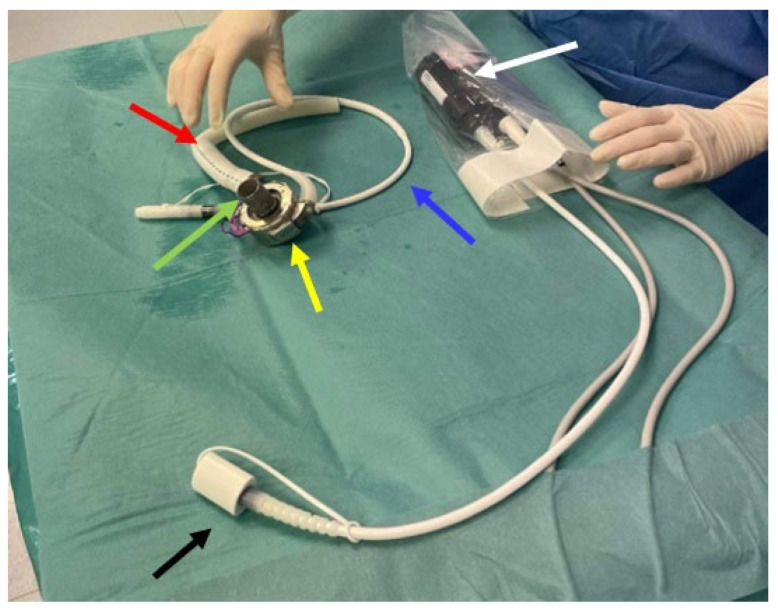
Basic components of an LVAD—Heart Mate III. Inflow cannula (green arrow); Pump (yellow arrow); Outflow cannula (red arrow); Driveline (blue arrow) Controller: (white arrow); Power source wire (black arrow).

LVADs serve to treat advanced heart failure and enhance cardiac output by aiding the ejection of blood from the left ventricle to the systemic circulation. The basic components comprising a LVAD are shown in Figure 1 and Figure 2. LVAD models from different manufacturers that are currently in clinical use contain variants of the following common functional components:

− Inflow cannula: a tube which draws blood from the left ventricle (apex) of the heart and delivers it to the pump (Figure 2, green arrow);
− Pump: the central component of the LVAD, assisting in pumping blood from the left ventricle of the heart to the circulatory system (Figure 2, yellow arrow);
− Outflow cannula: a tube that carries the pumped blood to the aorta (Figure 2, red arrow);
− Driveline (Figure 2, blue arrow): a percutaneous lead that connects the pump with an externally placed controller and power source (mobile battery system or stationary power and control unit (Figure 2, black arrow));
− Controller: the controller transmits information to the pump about the set rotational speed, measures the energy needed to operate the pump, and estimates the flow based on pump performance (Figure 2, white arrow).

**Figure 3:**
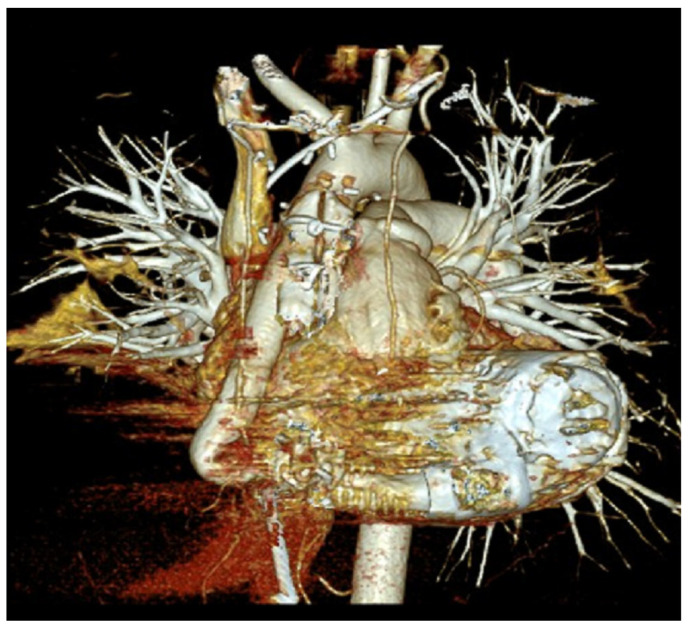
3D computed tomography reconstruction (PA view) of the heart and big vessels along with the LVAD system with its associated components in a patient with an implanted LVAD.

Modern LVADs are continuous-flow pumps with a central electromagnetically driven centrifugal rotor driving blood from an inlet cannula at the LV apex (Figure 1) to an outflow graft anastomosed with the ascending aorta [4]. Figure 3 presents a 3D computed tomography reconstruction (PA view) of the heart and big vessels relative to the locations of the LVAD components. Blood flow through LVADs is directly proportional to pump speed and inversely related to the pressure difference across the inlet (LV pressure) and outlet (aortic pressure) orifices of the pump [5]. The flow occurs consistently throughout the cardiac cycle.

### 1.2. Epidemiology of Ventricular Arrhythmias in LVAD Patients

Ventricular arrhythmias (VAS) is defined as episodes of sustained (>30 s) ventricular tachycardia (VT) or any episode of ventricular fibrillation (VF). Ventricular arrhythmias occur in from 20 to 50% of LVAD recipients and are the most significant cause of mortality [6,7]. The majority of ventricular arrhythmias in patients with LVADs are monomorphic, sustained ventricular tachycardia episodes with a re-entrant mechanism related to scar tissue (discussed further later in the article), accounting for up to 80% of cases. This is followed by ventricular fibrillation (VF) in 31% of cases, focal/micro-re-entry VT in 7%, and bundle branch re-entry VT in 3.5% of cases [8].

In Miller’s study, it was emphasized that VT more frequently occurs within 30 days from the date of implantation [8]. The occurrence of ventricular arrhythmias in the first week is associated with an increased risk of mortality [9,10,11]. Sacher et al. demonstrated that patients who had ventricular arrhythmias (VAs) within the first month after LVAD implantation also had a history of VAs before the procedure, suggesting that pre-existing VT is a significant risk factor for developing VT post-LVAD implantation, as confirmed by other studies [7,10,12,13]. Similarly, several papers have demonstrated that the risk of VAs following LVAD implantation is significantly lower when pre-existing VAs are absent (4% compared to 46%) [14].

Over time, the incidence of VT decreases, as confirmed by numerous observational studies [7,11,15]. Furthermore, the literature emphasizes an association between VT and ischemic cardiomyopathy in particular as the etiological agent of cardiac failure, which also constitutes the predominant cause of these arrhythmias in the general patient population [11,16]. Other independent risk factors of late ventricular arrhythmias are: preexisting atrial fibrillation, duration of cardiac failure exceeding 12 months, lack of treatment with angiotensin-converting enzyme inhibitors during follow-up [17]. 

Interestingly, patients with LVADs can tolerate VT or even ventricular fibrillation (VF) for days because of the hemodynamic support provided by the LVAD [18,19], even though “low flow” alarms may occur due to a poorly filled right ventricle. The Momentum 3 Study demonstrated that 6 months after LVAD implantation, VF was the cause of 1 out of 35 deaths during the observation period, and sustained VT requiring cardioversion or defibrillation was observed with notable frequency (20% axial flow pump vs. 18% centrifugal pump) [20]. Inadequately treated and long-standing ventricular arrhythmias may also lead to right ventricle failure [14,21]. The ASSIST-ICD study reported that within nine months of implantation, 10.7% of patients with LVADs experienced an electrical storm (ES), defined as three or more consecutive sustained episodes of ventricular tachycardia (VT), ventricular fibrillation (VF), or appropriate ICD shocks during a 24-h period. More than half of the patients with ES had their first episode during the first month, and 33% of patients died within 2 weeks of experiencing it [22].

### 1.3. Potential Mechanisms of Ventricular Arrhythmia in LVAD Patients

VAs most commonly occur in the early postoperative period, which is associated with, among other contributing factors, high sympathetic nervous system activity, fluid and electrolyte disturbances, as well as the use of inotropic medications [15,16]. In the first week after LVAD implantation, a transient change in repolarization in the form of QT interval prolongation was observed, potentially leading to an increased susceptibility to VT [23]. Additionally, the implantation of a cannula directly into the apex of the heart necessitates some unavoidable amount of cardiac muscle damage and the formation of scar tissue, which inevitably becomes a substrate for arrhythmias [15,16,24]. Sacher et al. observed that the primary substrate appears to be the intrinsic myocardial scar, rather than the apical cannula itself, with only 9% of the targeted VTs being associated with the LVAD cannula [12]. Similar findings were reported by Anderson et al., where scar-related re-entry was the prevailing mechanism for VT (90%), and VT related to the cannula occurred in 19% of cases [25].

An additional, rare mechanism of VT, accounting for 3% of cases, is connected to contact (suctioning) between the inflow cannula and the left ventricular wall [12]. This contact can occur via a wide variety of mechanisms. For example, excessive dehydration or even ventricular tachycardia itself can lead to a decrease in cardiac preload. This in turn can lead to a decrease in the effective volume of blood available to the pump, thus initially causing reductions in blood flow and triggering alarms. Furthermore, if a sudden and significant decrease in preload occurs, a device maintaining a constant rotational speed may cause further excessive volume unloading and become partially or fully suctioned to the walls of the left ventricle, arresting forward flow. This suction can, in turn, mechanically induce disturbances in the electrical conductivity and automaticity of the heart, leading to the onset of ventricular arrhythmias which further exacerbate the cycle of effective volume loss and device dysfunction [26]. Thus, ventricular arrhythmias may contribute to the disruption of LVAD function, and conversely, the LVAD itself under specific unfavorable hemodynamic conditions can be the cause of their onset.

## 2. Diagnostic Difficulties of Ventricular Arrhythmias in Patients with Left Ventricular Assist Device

**Figure 4:**
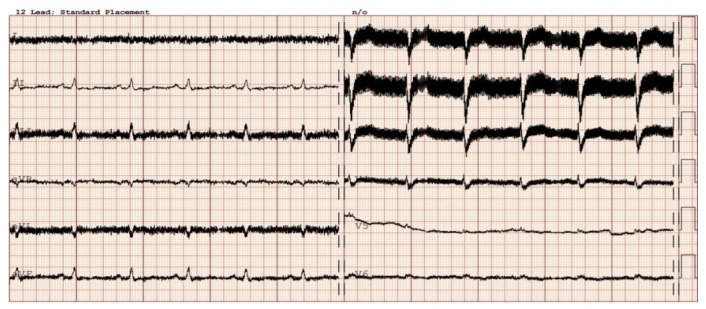
Sinus rhythm recorded in electrocardiography in a patient after implantation of a left ventricle assist device. Electromagnetic interference is visible in the majority of leads.

**Figure 5:**
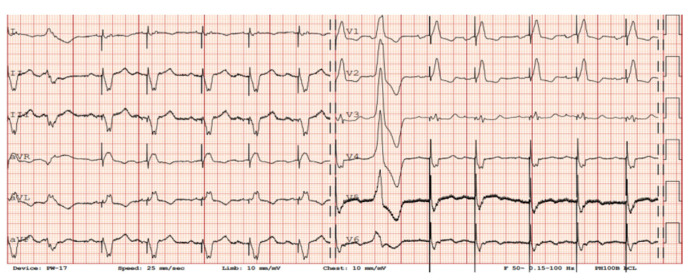
Sinus rhythm with sequential ventricular pacing (VAT mode), as well as a ventricular premature beat in patient with a LVAD. EM artifacts generated by the LVAD are visible in the III, V5–V6 leads.

**Figure 6:**
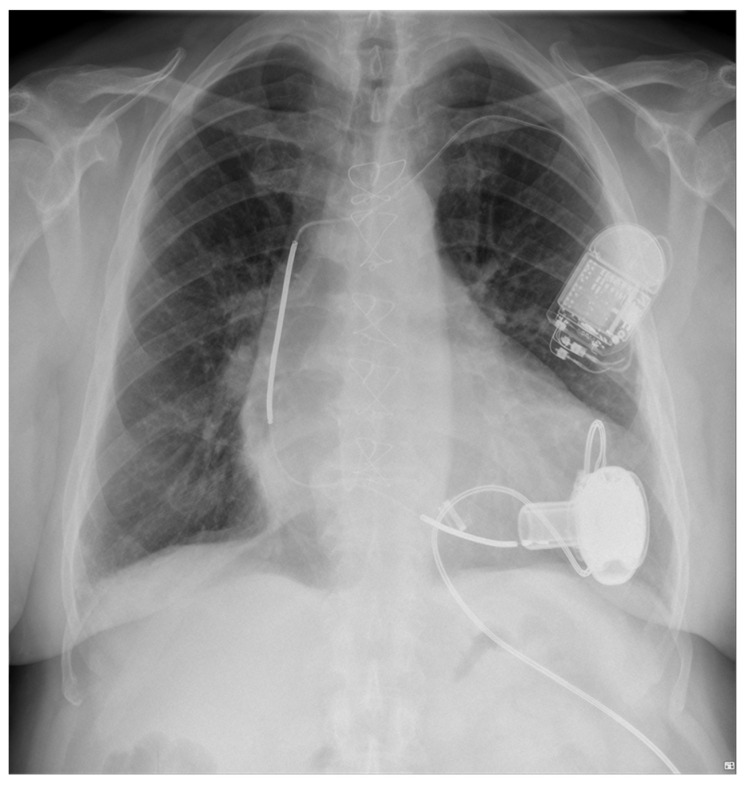
Chest X-ray. Posterior–anterior view. Patient with a transvenous single-chamber implantable cardioverter-defibrillator with a dual-coil lead and a left ventricular assist device. Very close proximity of the LVAD inlet cannula and the sensing bipole located on distal lead tip can be noted (structures overlapping).

Diagnosing VT in LVAD patients can be complex due to altered hemodynamics and device-related artifacts. Figure 4 and Figure 5 present examples of electrocardiographic traces in patients with an implanted LVAD. Common diagnostic tools include continuous remote monitoring (telemetry), 12-lead electrocardiograms (ECGs), and implantable device interrogation (if present). Continuous monitoring allows for real-time detection of arrhythmic events, while 12-lead ECGs can help identify VT morphology. Nevertheless, the electrical function of the heart may be monitored by simple examinations like a standard ECG [5], and it can provide additional valuable information about the heart rhythm. However, in patients with a LVAD, a standard ECG is often disturbed by the device’s magnetic interference. The localization of the rotor of the pump, as well as inflow and outflow cannula positioning, can also impact the presence of artifacts. Figure 6 presents a chest x-ray of a patient with a transvenous single-chamber implantable cardioverter–defibrillator (ICD) with a dual-coil lead and a left ventricular assist device, where the sensing bipole located on the distal lead tip of the ICD is located in very close proximity to the LVAD inlet cannula.

## 3. Electrocardiography in Patients with Implanted LVADs

### 3.1. Twelve-Lead ECG Findings in Patients with LVADs

**Figure 7:**
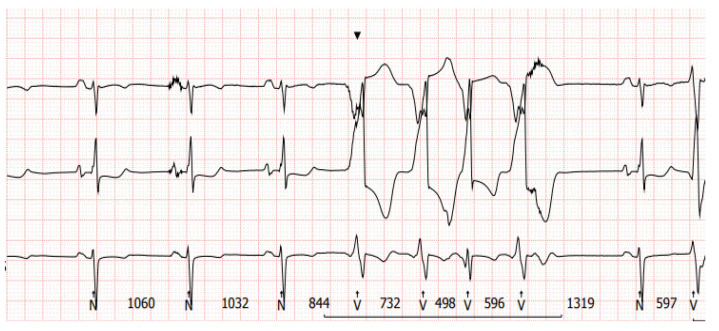
A 3-lead Holter recording in a patient with an implanted LVAD with a sinus rhythm and ventricular tachycardia. Transient interference at regular intervals can be seen in upper line lead.

**Figure 8:**
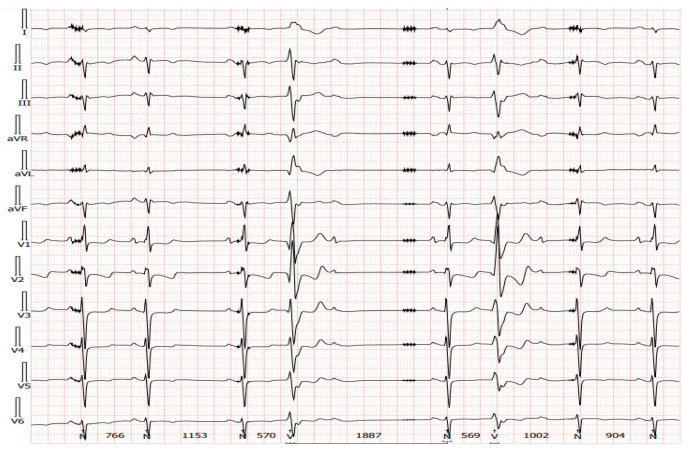
A 12-lead Holter recorded from the same patient as presented in Figure 7. Transient artifacts at regular intervals from the artificial pulse can be seen in most leads.

There are limitations to interpreting a standard ECG in this specific group of patients due to potential electromagnetic interference produced by the LVAD. Implantation of the device by itself may cause myocardial injuries, evoking ECG changes. Additionally, muscle fasciculations during device operation can induce additional artifacts [27,28]. Martinez SC et al. [29] categorized these relevant ECG changes in LVAD patients according to LVADS2: low limb-lead voltage, ventricular pacing, the electrical artifact duration of QRS complex, ST elevation in the lateral leads, and splintering of the QRS complex. Among them, electrical artifacts and low limb-lead voltage may have the strongest impact on proper ECG interpretation. In another study, the authors highlight additional general changes in ECG that occur after LVAD implantation, including the reduction in the amplitudes of the R and S waves in some leads and an alternation of the R:T ratio. These changes in baseline ECG as well as artifacts may have a significance in the proper functioning of implantable cardioverter–defibrillator devices (ICD), which we will discuss further in the article [27]. Interesting conclusions can be drawn from a study by Zak Loring et al. [30], in which it was shown that the use of dedicated filters at modified frequencies may contribute to the reduction in artifacts from cutaneous ECG in a group of patients with LVADs. The LVAD devices operate at a rotational speed within the range of 2400–3200 rpm (Heart Ware Medtronic) and from 5000 to 6000 rpm (HeartMate III Abbott), corresponding to oscillatory frequencies of 50–53.3 Hz and 83.3–100 Hz, respectively. It is worth considering their impact on the depiction of disturbances in the ECG. Furthermore, in the case of HM III, the device produces an artificial pulse every 2 s in order to prevent thromboembolic complications. This involves an acceleration of rotation by 2000 rpm followed by a deceleration, which is detectable in both three-lead (Figure 7) and twelve-lead Holter recordings (Figure 8) [31].

### 3.2. Alternative Diagnostic Methods of Noninvasive Electrocardiography in Patients with Implanted LVADs

**Figure 9:**
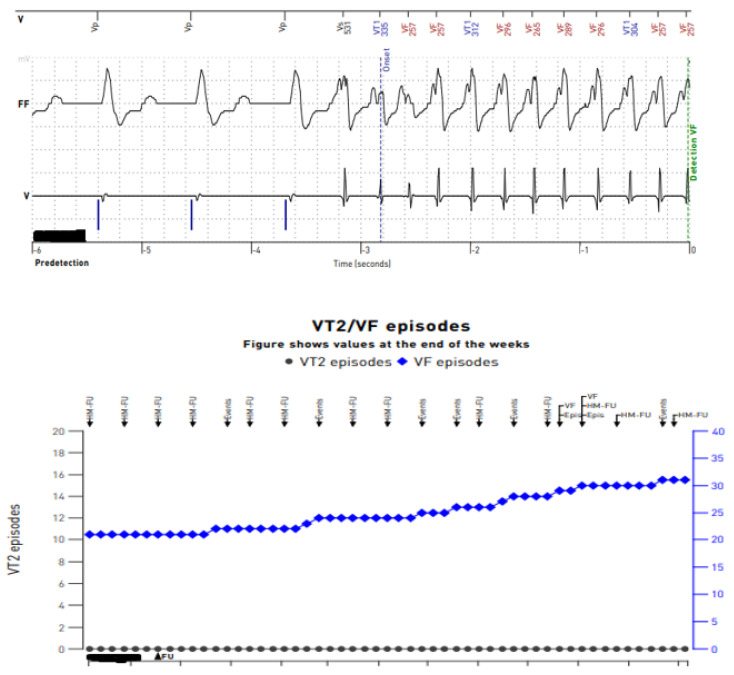
Reports from remote telemonitoring showing sustained ventricular tachycardia on an intracardiac electrogram recorded from transvenous leads of an implantable cardioverter–defibrillator.

In our daily clinical practice, we have observed a few different diagnostic methods that are particularly useful in recognizing arrhythmia origin in a LVAD patient. A 12-lead Holter is particularly high-yield. In contrast to standard ECG (Figure 4 and Figure 5), a 12-lead Holter monitoring (Figure 8), depending on the hardware and analysis software used, can provide tracings with minimal artifacts. A valuable diagnostic tool for assessing the incidence of arrhythmia is remote monitoring of an implanted cardioverter–defibrillator. In the reports sent by the remote systems, we can obtain precise information on the duration of arrhythmia, the number of episodes, delivered therapies, and the heart rate. An example of a graph from a ICD showing statistics regarding the number of therapies delivered, heart rate, and number of VT/VF episodes per day is presented in Figure 9.

## 4. LVAD and Cardiac Implantable Electronic Devices

Implantable cardioverters–defibrillators, both with and without resynchronization, make up the vast majority (approximately 50–90%) of CIEDs in LVAD patients. Across multiple published guidelines, ICD therapy is recommended in advanced heart failure patients who are candidates for LVAD or heart transplantation; however, its impact on survival in this group remains doubtful. Secondary rather than primary prevention actually demonstrates benefits in ICD therapy [19]. De novo ICD implantations in subjects previously equipped with a LVAD are still a subject of debate. ICD use in LVAD patients has also steadily decreased over the years [32].

### 4.1. Pulsatile vs. Continuous Flow in Terms of ICD Outcomes in Patients with LVADs 

Pulsatile LVADs were a dominant form of mechanical circulatory support in the previous decade. The majority of older data available (reaching together up to 2000 patients observed) show significant benefits of ICD use in pulsatile LVADs; specifically, its use was associated with a significant reduction in mortality in these patients with the rate of appropriate shocks being high though reduced vs. pre-LVAD periods [33,34,35]. The multicenter European PCHF-VAD registry of 448 patients with LVADs in which half (235 pts) were also implanted with an ICD showed that the ICD group had a 36% reduction in mortality in a multivariate analysis with an average follow-up time of 1.1 years. Any single VA incident in LVAD patients increased the risk of both all-cause and cardiovascular death, and an active ICD device resulted in its subsequent almost 50% reduction. The results support the conclusion that ICD implantation was associated with significantly better survival in LVAD patients regardless of when it was implanted [36].

In recent years, clinical practice has transitioned over to continuous flow devices. Galand V et al., in a multicenter observational analysis of 494 continuous-flow LVAD patients, identified predictors of VA. They proposed a VT-LVAD score with four risk groups stratified from 0–10 points each. This score could be useful in identifying patients, after the implantation of continuous-flow LVADs, who might benefit from subsequent ICD implantation [17]. Shockingly, compared to previous data, the Interagency Registry for Mechanically Assisted Circulatory Support registry with 2209 ICD patients and a propensity-score-matched group without the device, does not support the effectiveness and prognosis benefits offered by an ICD in continuous-flow LVAD recipients [36]. The presence of an ICD was actually associated with an increased mortality risk, heart transplantation (HTX) rate, number of VA hospitalizations, and no recovery with LVAD explantation. However, these data must be approached with caution, as the control group was selected on the basis of propensity score matching which could have been prone to confounding bias by latent covariables such as additional unknown common pre-existing conditions within the ICD group. In an observational INTERMACS group of 1444 different types of LVAD patients remaining on a waiting list for HTX and divided evenly between ICD and non-ICD subgroups, no benefits of ICD therapy were noted in total mortality, cardiovascular wait-list mortality, and HTX delisting in a median 5.6 month follow-up. However, there was a greater yet statistically insignificant incidence of death in the non-ICD population (five vs. two) [36]. Additionally, a meta-analysis of three observational studies with 203 patients (69.5%) with an ICD and continuous-flow LVAD, showed no benefit in terms of improved survival, severe right ventricular dysfunction, and LVAD-related complications [37,38].

### 4.2. ICD Implantation Recommendations for Patients with LVAD

Because current long-term circulatory support devices are mostly continuous-flow systems, based on the above, the available data only support the use of de novo ICDs in those patients with a cautious and individualized approach, even as VAs remain a significant clinical challenge. Therefore, the 2022 ESC guidelines for the management of patients with ventricular arrhythmias and the prevention of sudden cardiac death recommend that an ICD should be considered in LVAD patients with symptomatic sustained VA (strength of recommendation IIa/quality of data B). One should also note that therapeutic options such as ablation may be considered within a comprehensive approach. Over the coming years, increasing survival time of LVAD patients, especially with the ever-increasing popularity of LVADs as destination therapy, might lead to a reassessment of the role of CIEDs in this patient population. Theoretically, wearable cardioverter–defibrillators could be an alternative in selected cases of advanced or end-stage heart failure, but due to a limitation on the duration of their use and possible interference with LVADs, this method is currently rather limited to patients on the urgent waiting list for HTX who have not previously been implanted with an ICD [39].

### 4.3. Technical Considerations in ICD Programming in Patients with LVADs

Optimal device programming (including but not limited to pacing modes, CRT function, and ICD shock thresholds) is crucial for proper hemodynamics and ventricle performance. Additionally, with ICD shocks, “too aggressive” programming with discharges may impair quality of life, while more conservative programming may not induce ICD shocks when necessary [40,41,42]. It is also worth noting the potential for electromagnetic interference (EMI) issues upon the LVAD device with inappropriate shocks. 

In regard to the strategy of pacing programming, the latest evidence may support switching off left ventricle pacing altogether in LVAD patients with previously implanted CRTs. Furthermore, as Chung BB et al. have shown in a randomized crossover study, in 30 LVAD patients with CRT, RV pacing was associated with only functional status and quality-of-life improvement alongside a reduction in ventricular arrhythmias as compared to the CRT-only control group [43]. On the other hand, there are data available that support the superiority of biventricular pacing over other pacing modes in acute improvement of RV contractility in patients with LVADs [44]. Clearly, there is much room available for personalized management of the ventricular pacing strategy in LVAD patients.

**Figure 10:**
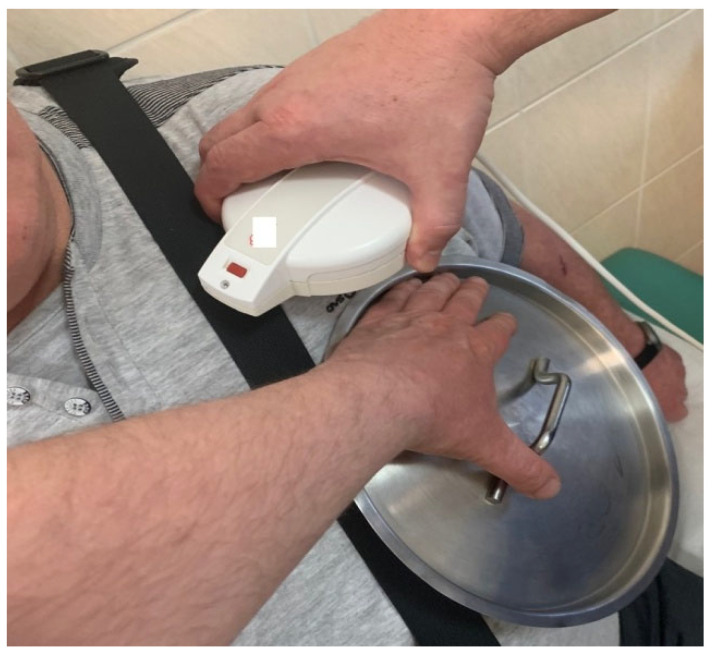
CIED interrogation in a LVAD patient. Presented is the “Metal lid” (or “frying pan”) technique. Here, the metallic object serves as a pseudo Faraday cage (a conductive cage made of metal aimed to shield against electromagnetic and electrostatic fields) above the LVAD in order to protect the programmer’s head from any potential interference.

Vastly increasing numbers of various implantable therapeutic devices and external diagnostic devices are generating a clinical problem of potential EMI. External sources of EMI have been listed on the safety instructions for a long time and any new device or electric tool combination must be taken into account with respect to its clinical relevance. The main concern in cardiology is the use of defibrillation devices (ICD). LVADs, due to the proximity of an ICD lead-sensing dipole, may cause EMI noise detection and inadequate device discharges, which has been the subject of many studies and observations [45,46,47,48]. The coexistence and proximity of ICD and LVAD devices is shown in Figure 10. 

The incidence of LVAD-related noises has been found to be significantly high in S-ICD patients, which affects implantable defibrillator function. Incidence may extend from 15% up to 33%, and HeartMate III devices were prone to interfere with S-ICDs, as in the presented case [47]. There were cases where turning off the device must have been executed as the only option to eliminate painful inappropriate shocks. The importance of awareness of such device interference should be highlighted, and this indicates the need for further algorithm improvements to eliminate this phenomenon. Novel extended device-based S-ICD screening methods have been developed for LVAD subjects as well, due to a small number of S-ICD-eligible LVAD patients and screened with the standard test to eliminate EMI [48]. Modified and robust screening should be recommended in patients with LVAD who are candidates for S-ICD therapy.

Additionally, EMI which may seriously interfere with cardiac pacemakers can be generated by deep brain stimulation devices. In regard to transvenous and leadless pacemakers, clinical experience and observational data are still limited due to the rarity and novelty of the technologies. Their potential for EMI interactions with LVADs is yet to be explored [49].

## 5. Medical Therapy in Patients with LVAD and Ventricular Arrhythmias

According to the available data, even though a LVAD may have sufficient hemodynamic output even in sustained ventricular arrhythmias (utilizing passive right ventricle filling and pulmonary flow), ventricular arrhythmias should still be treated actively, as they may result in a worsening clinical outcome and cause higher mortality [9]. However, this opinion is not unanimous. Gulleta S et al. have shown that ventricular tachyarrhythmia has not affected mortality, and furthermore, that ICD implantation has not demonstrated an impact on survival [42].

In the management of VA in patients with LVADs, a range of therapeutic approaches is at our disposal, paralleling those employed in heart failure management [1]. The existing protocols are commonly derived from research focused on the administration of antiarrhythmic drugs to VAs in patients diagnosed with structural heart disease and equipped with ICDs [45]. Data regarding the optimal management of VA are limited and primarily derived from small observational studies and case reports [7,11].

After LVAD implantation, patients experiencing new ventricular arrhythmias should undergo prompt evaluation for hemodynamic stability, utilizing the currently available hemodynamic monitoring tools, as well as be electrically monitored with ECG. In the presence of hemodynamically unstable VT, the recommended initial intervention is prompt electrical cardioversion [50,51]. In the case of ventricular fibrillation (VF), immediate defibrillation is indicated. Furthermore, echocardiographic examination should be performed to exclude episodes of left ventricular volume unloading leading to suction by the device on the ventricular wall, as mentioned previously in the article. If such an event is detected, appropriate adjustments to the pump settings (most commonly, a reduction rotational speed and flow) should be made to optimize device performance [12,15,51].

In stable patients, the diagnostic approach should be extended to include the most common causes of cardiac arrhythmias, such as electrolyte abnormalities. Also, patients may require oral or intravenous administration of antiarrhythmic medications such as amiodarone or beta-blockers [50,51]. In the short-term setting, the preferred drug regimen remains intravenous amiodarone and sodium channel-blocking agents such as lidocaine and procainamide. For long-term management, additional pharmacological options for VA suppression include mexiletine and dofetilide [52]. 

Considering that amiodarone is a popular and frequently used antiarrhythmic medication, it is essential to note that it does not inherently increase the risk to survival in this patient group [49]. In fact, none of the currently available drugs have demonstrated a reduction in mortality rates compared to an ICD [53]. Instead, amiodarone may alleviate symptoms and potentially reduce ICD interventions. However, its numerous side effects require frequent clinical monitoring. Mexiletine, often used in cases of amiodarone ineffectiveness, has actually been found to be relatively less effective in study by Rasch [7]. 

## 6. Catheter Ablation in Patients with LVADs and Ventricular Arrhythmias

Ventricular arrhythmias can exert a notable influence on the prognosis of patients with LVADs. Catheter ablation might be an effective option for VT refractory to medical therapy [54,55]. The process of mapping and ablation of arrhythmias may present challenges due to mechanical and electromagnetic constraints. A meticulous pre-procedural plan can play a pivotal role in diminishing the occurrence of complications [55].

In 2007, Dandamudi et al. reported the first trials of VT ablation. Since then, many papers have presented very good results of ablation in LVAD patients [49]. In total, however, the literature contains only a few series of cases and limited observational studies on a few dozen subjects with LVADs referred for ablation [25,56]. The majority of procedures performed were endocardial with the preexisting scar as the main target. Ablations targeting a cannula-related substrate make up a minority of cases. The effectiveness of ablation in periprocedural or short-term observation varies between 77% and 86%; however, its impact on survival is low in this high-risk population. The presence of LVAD hardware makes the procedure more complex but does not increase specific mechanical adverse events. RF ablation in LVAD patients is, however, associated with a higher rate of thromboembolic complications [57]. Three-dimensional mapping systems and intercardiac echocardiography may enhance precision during ablation, thereby reducing the risk of complications. A study to evaluate the use of prophylactic intraoperative ablation in LVAD patients and its effects on prognosis and complications is planned [58].

While not routinely utilized in practice, as can be seen from the totality of published reports, the most commonly accepted indication for catheter ablation in LVAD patients is incessant VT, electrical storm, recurrent ICD interventions, or progressive RV failure due to arrhythmia. Performing either endocardial or epicardial ablations during implantation has proven to be a safe approach, leading to a significant decrease in the arrhythmic burden after the procedure, especially when ventricular arrhythmias were recurring before surgery. The combination of epicardial with endocardial ablations yields better outcomes and offers exceptional visualization of the ablation site, surpassing what can be achieved with endovascular methods [8]. 

### 6.1. Ablation Post-LVAD Implantation: Technical Considerations

The techniques of catheter ablation are similar to those used in patients without a LVAD, with a few additional considerations. It is essential to maintain anticoagulation without interruption during ablation in LVAD patients to mitigate the substantial risk of thromboembolism. The use of ultrasound-guided canulation of vessels has become standard due to a lower risk of adverse events. 

The apical placement of the LVAD pump and the suction force exerted by the inflow cannula represent a risk factor for inadvertent catheter insertion into the impeller. However, it is worth noting that, up to this point, no instances of such a complication have been reported. Furthermore, in order to avoid interference with the outflow cannula, a transseptal puncture may be considered as a preferred method to access the left ventricle. Difficulties may arise with transaortic access due to reduced flow through the aorta proximal to the outflow tract, leading to reduced or even absent natural opening of the aortic cusps, potentially increasing the risk of insertion failure and/or thromboembolic events. Epicardial mapping and ablation, which may become necessary in certain cases, present challenges due to the restricted pericardial space, the surgical placement of the LVAD, and the potential risk of damaging parts of the device. Additionally, infection risk is increased due to the presence of artificial materials within the pericardial space and the left ventricle.

Standard practice involves utilizing a three-dimensional electro-anatomic mapping system for substrate mapping. The 3D system is employed to identify areas of interest, as well as regions with low voltage and scar tissue. The induction of ventricular arrhythmias (entrainment) can be utilized to map ventricular tachycardia. Entrainment mapping enables the diagnosis and characterization of re-entrant arrhythmias from an analysis of the interaction between pacing maneuvers and resulting tachycardia. In LVAD patients, entrainment maneuvers are feasible due to the fact that VT may be even better tolerated from a hemodynamic standpoint. Macro-reentrant ventricular tachycardia is recognized by its activation pattern, and subsequently, entrainment techniques are performed to demonstrate that the arrhythmia of interest is isthmus-dependent. Focal ventricular tachycardia or micro-reentrant ventricular tachycardia is diagnosed when the activation pattern is concentric. It is also important to note at this point that the electromagnetic interference emanating from a LVAD can be a significant issue during map acquisition, as mapping systems rely on magnetic fields to create three-dimensional maps. However, Anderson reported that electro-anatomical mapping interference occurred in only 1.8% of cases [25]. Furthermore, Vaidya et al. noted significant electromagnetic interference (EMI) in 9.4% of all mapping points attempted when using Carto3 at the LVAD’s maximum performance level. Notably, there was no occurrence of severe EMI during impedance-based mapping [59,60].

In order to provide the anatomical data when performing mapping, pre-procedural CT with 3D reconstruction of the left ventricle and the cannula, or intracardiac echocardiography (ICE), can be used. Compared to static CT images, intracardiac echo provides real-time imaging of cardiac structures, aiding in the precise localization of arrhythmogenic foci and improving procedural outcomes [10]. The combination of intracardiac echo with electro-anatomical mapping systems has been shown to improve the accuracy of substrate identification in LVAD patients with VT. By providing a comprehensive view of intracardiac structures alongside electrophysiological information, ICE facilitates the creation of detailed 3D maps, optimizing ablation target selection and reducing procedure times [11]. 

Given the altered anatomy and continuous blood flow support provided by LVADs, safety is paramount during ablation procedures. Several studies have demonstrated the utility of ICE in visualizing the LVAD inflow cannula, outflow graft, and adjacent structures, enhancing the safety and efficacy of catheter ablation [61,62,63]. Intracardiac echo aids in the visualization of potential complications, such as catheter entrapment or interference with LVAD components, minimizing the risk of adverse events [61]. Additionally, ICE enables real-time monitoring of cardiac function, ensuring the preservation of proper LVAD function throughout the ablation procedure [61]. Studies evaluating the impact of intracardiac echo on clinical outcomes in VT ablation in LVAD patients are limited but encouraging. Improved procedural success rates, reduced recurrence of VT, and enhanced patient safety have been reported in cohorts where ICE was integrated into the ablation strategy [61,62,63]. While further research is needed to establish standardized protocols and assess long-term efficacy, current evidence supports the routine use of intracardiac echo in VT ablation for LVAD patients.

### 6.2. Results of Catheter Ablation of Ventricular Tachycardia in LVAD Patients

Ablation of VTs in patients with LVADs should be considered if there are indications for the procedure given that acute procedural success, complications, and recurrence rate are comparable to the non-LVAD patient groups [8]. Moss et al. reported that, among surviving patients, freedom from recurrent VT was 64% at 1 year, with an overall 1-year survival rate of 67% for patients without an arrhythmia recurrence and 29% for those with recurrence [59]. In the Cantillon group, catheter ablation was successful in 18 out of 21 patients (86%). VT recurred in 7 of 21 patients (33%) at an average of 133 ± 98 days, and 6 patients (29%) required repeat procedures, with subsequent recurrence in 4 out of 21 patients (19%) [24]. Similarly, Sacher et al. concluded that despite an overall high mortality rate among left ventricular assist device recipients, catheter ablation of ventricular tachycardia is effective and relatively safe even within the first month following implantation. They found that the intrinsic myocardial scar, rather than the apical cannula, appears to be the primary substrate for arrhythmia formation [12].

## 7. Summary

Heart failure treatment using left ventricular assist devices is challenging for specialists in many fields. The cooperation between the patient and multidisciplinary team of heart failure specialists, cardiologists, cardiac surgeons, electrophysiologists, nurses, perfusion specialists, and VAD coordinators among others is particularly important to achieve the highest effectiveness of treatment and reduce the occurrence of complications. One of the situations that requires immediate (but tailored to the patient’s individual needs) intervention is the occurrence of ventricular arrhythmias. VTs are generally well tolerated in patients with LVADs; however, their occurrence may affect prognosis and the effect of a wide variety of therapeutic interventions. Due to the presence of interference from the LVAD’s electromagnetic field, the diagnostic process using electrodiagnostic methods may be difficult. In doubtful situations, it is worth considering the parallel application of various ECG recording methods from varied sources—both the standard 12-lead ECG recording and Holter continuous recording, in addition to any EGM recordings from implanted electrotherapy devices and even from wearable devices (smartwatches, etc.). Upon diagnosis of arrhythmia, optimal pharmacological, electrical, and/or invasive management should be tailored to the patient’s individual needs, clinical presentation, and preferences. When ventricular arrhythmias are well tolerated by patients on LVAD therapy, treatment should not be too aggressive, and in the case of a stable patient, treatment should primarily rely upon pharmacological and less invasive interventions with a comprehensive evaluation of the underlying causes of arrhythmia. According to historical data, utilization of ICDs is associated with significantly better survival in pulsatile LVAD patients; however, due to recent mixed findings in the continuous-flow LVAD population, de novo implantation of an ICD in a LVAD patient should still be considered with caution with the final decision individualized to each patient. Notwithstanding the contradictory evidence in the literature for ICD use in LVAD patients presented in this article, according to our experience, therapy with ICD devices should still be considered, given the improvement to quality of life and psychosocial functioning. Regardless of current ICD implantation status or candidacy status for future ICD implantation, end-of-life care must still be equally and independently considered in all LVAD patients.

Finally, catheter ablation of ventricular tachycardia in LVAD patients has been demonstrated to be effective and relatively safe and should be considered in patients with recurrent, hemodynamically significant ventricular arrhythmias. 

## Data Availability

All data underlying the results are available as part of the article and no additional source data are required.
